# The Dynamics of Histone Modifications during Mammalian Zygotic Genome Activation

**DOI:** 10.3390/ijms25031459

**Published:** 2024-01-25

**Authors:** Francisco Sotomayor-Lugo, Nataly Iglesias-Barrameda, Yandy Marx Castillo-Aleman, Imilla Casado-Hernandez, Carlos Agustin Villegas-Valverde, Antonio Alfonso Bencomo-Hernandez, Yendry Ventura-Carmenate, Rene Antonio Rivero-Jimenez

**Affiliations:** Abu Dhabi Stem Cells Center, Abu Dhabi P.O. Box 4600, United Arab Emirates; francisco.s@adscc.ae (F.S.-L.); nataly.barrameda@adscc.ae (N.I.-B.); yandy.castillo@adscc.ae (Y.M.C.-A.); imilla.hernandez@adscc.ae (I.C.-H.); carlos.villegas@adscc.ae (C.A.V.-V.); antonio.bencomo@adscc.ae (A.A.B.-H.); yendry.ventura@adscc.ae (Y.V.-C.)

**Keywords:** histone modifications, chromatin landscape, mammals, embryonic development, embryonic stem cells

## Abstract

Mammalian fertilization initiates the reprogramming of oocytes and sperm, forming a totipotent zygote. During this intricate process, the zygotic genome undergoes a maternal-to-zygotic transition (MZT) and subsequent zygotic genome activation (ZGA), marking the initiation of transcriptional control and gene expression post-fertilization. Histone modifications are pivotal in shaping cellular identity and gene expression in many mammals. Recent advances in chromatin analysis have enabled detailed explorations of histone modifications during ZGA. This review delves into conserved and unique regulatory strategies, providing essential insights into the dynamic changes in histone modifications and their variants during ZGA in mammals. The objective is to explore recent advancements in leading mechanisms related to histone modifications governing this embryonic development phase in depth. These considerations will be useful for informing future therapeutic approaches that target epigenetic regulation in diverse biological contexts. It will also contribute to the extensive areas of evolutionary and developmental biology and possibly lay the foundation for future research and discussion on this seminal topic.

## 1. Introduction

Mammalian fertilization initiates with the fusion of an oocyte and a single sperm, a critical event in which these two terminally differentiated germ cells must undergo reprogramming to establish a totipotent zygote [1,2,3]. While germ cell chromatin is globally transcriptionally silent before fertilization, particularly regarding mRNA transcription, it is noteworthy that several non-coding RNAs (ncRNAs) are nascent expressed, playing critical roles in meiosis and post-fertilization development [4]. The oocyte’s meiotic resumption and the early zygote’s development rely on stored maternal transcripts until these are gradually degraded. The transition, known as the maternal-to-zygotic transition (MZT) [5,6], involves the meticulous coordination of maternal product clearance with zygotic genome activation (ZGA), marking the initiation of transcriptional control and gene expression post-fertilization [7,8]. As this intricate transformation unfolds, control over developmental processes gradually shifts to the RNAs and proteins newly synthesized in the zygote. It becomes evident that epigenetic modifications are pivotal in orchestrating this fundamental transformation [9]. Subsequently, ZGA is succeeded by the emergence of distinct cell identities within embryonic cells, leading to their differentiation into the inner cell mass (ICM) and trophectoderm (TE) stages at the blastocyst stage [10].

Epigenetic modifications occurring in terminally differentiated gametes, including DNA methylation [11,12,13], histone modifications [14,15,16], chromatin accessibility [17,18,19], and 3D chromatin structures [20,21], can be reset to a foundational state following fertilization. This reset process is crucial for achieving totipotency and supporting the subsequent development of a new individual. The precise regulation of zygotic gene transcription is intricately linked to chromatin accessibility. It underscores the pivotal role of epigenetic information in upholding cellular identity and governing gene expression. The nucleosome, serving as the fundamental unit of chromatin, consists of octamers comprising two copies of the core histone proteins H2A, H2B, H3, and H4 [22,23]. The modulation of chromatin accessibility is mediated via the positioning and configuration of nucleosomes, factors influenced by histone variants, and the post-translational modification of histone N-terminal tails. Several studies have increasingly suggested that histone modifications and variants are pivotal in ensuring precise control over ZGA [24,25,26].

Recent advancements in low-input chromatin analysis technologies have introduced innovative methods to address the challenges associated with the inaccessibility of early-stage embryos [3]. These breakthrough approaches have enabled a comprehensive investigation of the epigenetic remodeling mechanisms at the whole-genome level. In this review, our primary goal is to provide an in-depth exploration of the recent advancements in our understanding of the dynamic changes in histone modifications during the activation of the zygotic genome in mammals. These considerations will be useful for informing future therapeutic approaches that target epigenetic regulation in diverse biological contexts.

## 2. Methods

To compile this review, we conducted a comprehensive search of the Google Scholar and PubMed databases for relevant references from October up to December 2023 using the following terms: “epigenetics”, “histone modifications”, “chromatin landscape”, “mammals’ embryonic developments”, and “embryonic stem cells”. To describe detectable global histone patterns in gametes and early embryos, we based our review on analyses conducted by various authors. Our literature review primarily focused on genome-wide distribution patterns determined by state-of-the-art genomics approaches such as DNase-seq, ChIP-sequencing (ChIP-seq), ATAC-sequencing (ATAC-seq), and array hybridization. A limited number of studies that addressed global patterns via immunostaining and fluorescence microscopy were reviewed. Also, the cited bibliography of each selected paper was revised to identify any further relevant publications that aligned with our search objectives. The full text of each article included in this review was examined. Finally, we selected only the articles written in English and Spanish.

## 3. Comprehensive Overview

ZGA is not a singular event but a period during which transcription gradually becomes activated, marked by two distinct transcriptional waves. The first, smaller wave occurs during the early cleavage divisions, while the second, more significant wave coincides with the pause in the first division cycle across diverse species [4,27]. Although the precise timing of these waves and the number of division cycles vary among species [28], the process within a given species is meticulously controlled, exhibiting highly reproducible temporal patterns. Species with rapid development, such as worms [29], frogs [30], fish [31,32], and flies [33,34], complete the MZT and enter gastrulation only hours after fertilization. In contrast, in mammals with a more prolonged development, such as mice [35,36] and humans [37,38], MZT takes one or more days. This disparity is believed to stem from the egg’s nature, suggesting that each egg’s unique requirements dictate different embryogenesis modes [39]. Despite these variations, fundamental processes are conserved, and in all animals, the precise onset of ZGA relies on intricately coordinated mechanisms [4]. In this context, it becomes evident that diverse regulatory mechanisms orchestrate gene expression to establish and define cellular identity and fate.

Among these regulatory mechanisms, the dynamic processes of promoting or removing methylation [24], acetylation [40], phosphorylation [41], SUMOylation [42], and ubiquitination [43,44] marks on histones actively participate in chromatin modification during ZGA. Histone modifications, particularly methylation and acetylation, are crucial in regulating transcription by altering chromatin structure and providing binding platforms for transcription factors and other regulators [45,46].

Comparing genome activation across diverse mammals unveils common occurrences [32]. Following fertilization in mice, pronuclei harbor the genetic material from the egg and sperm, which remain separate in the zygote. The first embryonic transcripts emerge in the male pronucleus during the G2 phase of the initial cell cycle [47]. Subsequently, the pronuclei fuse, triggering the first embryonic division a few hours after fertilization [48]. Transcription in the 2-cell stage, dependent on RNAPII, is essential for subsequent cell division. During the transition from 4 to 8 cells, the second transcriptional phase marks the onset of morphological changes leading to blastocyst formation. In the 8-cell stage, blastomeres undergo compaction, followed by the specification of cell fates into ICM and TE [49]. This period culminates in cavitation, where fluid accumulates between both cell masses. Morphological similarities in early embryonic development between humans and mice, such as pronuclear fusion, cleavage divisions, compaction, morula formation, and cavitation, are evident. However, notable differences in genome activation and other morphological changes stand out. In humans, ZGA accelerates between the stages of 4 to 8 cells instead of 2 cells, as in mice. The significant presence of the transcriptional cohort of thousands of genes mediated by RNAPII in the 4- to 8-cell stage is crucial for subsequent cell divisions [19]. These later cellular stages resemble those in other mammals, suggesting that early human development is representative of mammals in general.

Histone methylation occurs on specific lysine and arginine residues of these proteins without altering their electrical charges [50]. Depending on the methylated residue, methylation can have diverse effects on gene transcription, either activating or repressing it [51]. In contrast, histone acetylation is closely associated with active gene transcription. This process, highly enriched at the transcription start site (TSS), involves changing the charge of lysine residues from basic to neutral. Histone acetylation has the effect of unpacking chromatin structure, increasing its accessibility for transcription processes [52].

The composition of a unique set of histones and their variants in the nucleosome is instrumental in loosening chromatin structure during ZGA. The subsequent sections delve into the specific roles of some of the most studied methylation and acetylation modifications in histone proteins, shedding light on their contributions during this critical developmental period.

## 4. H3K4me3 in Zygotic Genome Activation and Gene Expression Regulation

Histone H3 Lysine 4 trimethylation (H3K4me3) is an epigenetic modification associated with packaging DNA in eukaryotic cells, including human cells. It involves adding three methyl groups (trimethylation) on lysine residue at position 4 of the histone H3 tail. This modification plays a crucial role in regulating gene expression by altering the accessibility of genes for transcription [53,54]. H3K4me3 is commonly linked to the activation of nearby gene transcription. It achieves this by facilitating chromatin remodeling via interactions with the Nucleosome remodeling factor (NURF) complex [55]. This process enhances the accessibility of DNA for transcription factors, enabling genes to be transcribed and expressed within the cell. Beyond its role in gene expression, H3K4me3 is a key player in genetic regulation related to stem cell potency and lineage determination [56]. It is predominantly found in DNA regions associated with development and the establishment of cell identity. This epigenetic modification contributes to the intricate control of gene expression, ultimately influencing cellular functions and fate.

### 4.1. H3K4me3 at Promoters and Across Species

H3K4me3 is a canonical activation mark typically present at gene TSSs [57] and exhibits variations among species [4]. In zebrafish, H3K4me3 is detected at many promoters before ZGA, priming genes for activation [31,57,58]. Similarly, H3K4me3 is observed in frogs before genome activation, increasing during gastrulation [59]. In contrast, in *Drosophila*, only a few promoters exhibit H3K4me3 before the major wave of ZGA, implying that early transcription during the minor wave can proceed without this chromatin signature [60]. Nonetheless, a substantial upsurge in H3K4me3 accompanies the major wave of transcription in zebrafish, frogs, and *Drosophila*. In mice, early embryos exhibit broad domains of H3K4me3, which later become predominantly associated with conventional TSSs. Notably, depletion of the demethylases responsible for this shift leads to developmental arrest and downregulation of numerous ZGA genes [61].

In humans, signals of H3K4me3 can be identified at each stage of oocyte and embryonic development. The levels of H3K4me3 gradually decline from the germinal vesicle (GV) to the metaphase II (MII) stage and increase from the zygote stage to the four-cell stage, reaching their lowest point at the eight-cell stage [62]. A notable surge is then observed at the blastocyst stage. Notably, it is important to highlight that H3K4me3 in human oocytes is linked to CpG-rich regions and significantly correlates with CpG density [63]. This association appears to be a species-specific feature, pivotal in marking genomic regions preferentially activated during the MZT.

### 4.2. Dynamic Reprogramming of H3K4me3

The transition from specialized and transcriptionally silent gametes to a totipotent and transcriptionally active embryo involves a comprehensive reorganization of chromatin in the zygote. Thanks to recent advances in low-input ChIP-seq techniques, researchers have extensively explored the genome-wide landscape of H3K4me3 during ZGA and the first cell fate decisions in early embryos [64,65]. This process, initiated after fertilization, has been most studied in mouse embryos and entails the replacement of protamines with histones supplied by the mother. It explains why, after fertilization, H3K4me3 peaks of the paternal genome rapidly depleted in the mice zygote, revealing significantly different patterns compared to sperm. This phenomenon suggests extensive post-fertilization reprogramming, [66] with the swift exchange of protamines establishing a chromatin state that sets the ZGA stage.

**Figure 1:**
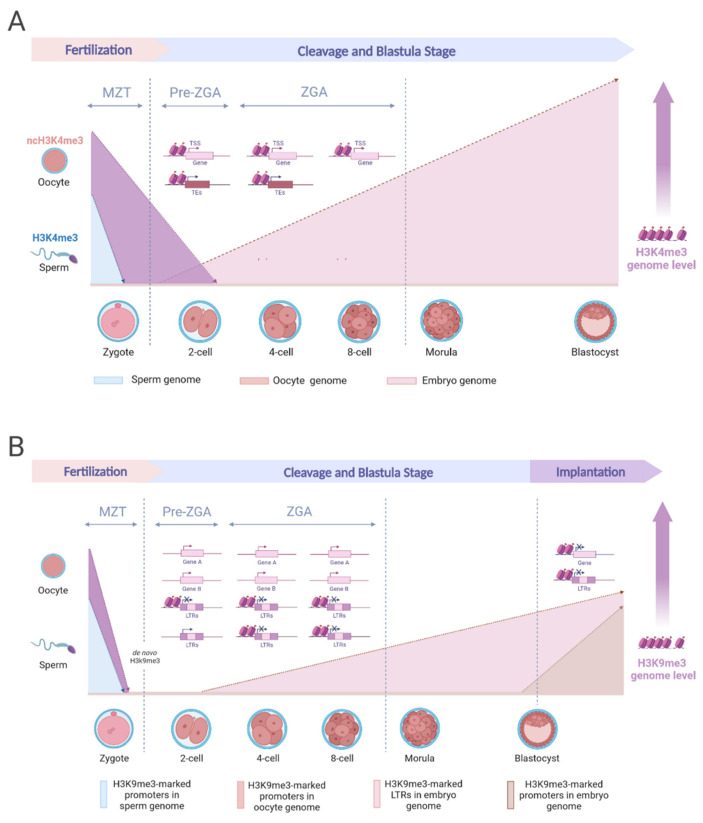
Epigenetic dynamics of H3K4me3 and H3K9me3 in early mouse embryogenesis. (**A**) H3K4me3 experiences swift depletion in the paternal genome post-fertilization, only to be reinstated during ZGA. Conversely, the deposition of ncH3K4me3 coincides with the silencing of the genome originating from fully grown oocytes and is subsequently substituted by canonical H3K4me3 at the 2-cell stage. Throughout ZGA, chromatin accessibility becomes evident at the H3K4me3-marked TSS sites of active genes. Simultaneously, transposable elements exhibit enrichment in H3K4me3. Progressing through development, there is a gradual escalation of H3K4me3 marks, culminating in forming a bivalent state with H3K27me3, priming the activation of developmental genes during the blastocyst stage. (**B**) Maternal genome-specific regions with H3K9me3 marks are more prevalent than paternal genome regions during early embryogenesis. The onset of early embryonic development triggers extensive DNA demethylation, resulting in the hypomethylation of actively transcribed regions, including LTRs. After fertilization, H3K9me3 marks within LTRs gradually increase during pre-implantation development. The majority of parental H3K9me3 regions are established de novo after fertilization. H3K9me3-enriched LTRs progressively emerge after the 4-cell stage and play a role in LTR silencing. Additionally, H3K9me3 marks in the promoters of developmental genes are erased after fertilization and restored after implantation. Abbreviations: LTRs, long terminal repeats; MZT, Maternal-to-zygotic transition; TEs, transposable elements; TSS, transcriptional start sites; ZGA, zygotic genome activation. Created with www.biorender.com (accessed on 25 December 2023).

Consequently, robust peaks of paternal H3K4me3 are re-established from the late two-cell stage onward [67]. Conversely, the maternal genome contains a noncanonical form of H3K4me3 (ncH3K4me3), covering broad domains in both promoters and distal regions [68]. NcH3K4me3 is already established in mature oocytes (MII), unlike in growing oocytes, where H3K4me3 maintains a conventional pattern of narrow peaks at the gene promoters. The noncanonical form of H3K4me3 is deposited during oocyte maturation and remains unaltered by canonical form until the major ZGA stage. The initiation and elimination of ncH3K4me3 represents a distinctive trait observed in the genomes of oocytes and early embryos [61,69]. Its removal is a crucial process for normal ZGA (Figure 1A). The deposition of the ncH3K4me3 coincides with genome silencing from fully grown oocytes to the early 2-cell stage [56,70]. The underlying maternal factors within the oocyte cytoplasm are pivotal in orchestrating the rapid change in the H3K4me3 landscape. Identifying these key maternal factors holds the potential to shed light on the mechanisms of H3K4me3 remodeling.

Broad H3K4me3 domains in both promoters and distal regions are actively removed by lysine demethylases KDM5A and KDM5B [71,72]. These enzymes allow the removal of ncH3K4me marks and the resetting of canonical H3K4me3 peaks until the two-cell stage [73]. The absence of Kdm5b results in the extension of the broad ncH3K4me3 domains, disrupting precise lineage differentiation [74]. On the other hand, the overexpression of Kdm5b leads to transcriptome reactivation in mature oocytes, hinting at ncH3K4me3’s potential role in genome-wide silencing. Moreover, transposable elements such as B1/B2/B4 and ERVL overlap significantly with ncH3K4me3 in distal regions [75], suggesting a correlation between ncH3K4me3 and repeat activities [68]. Nevertheless, the prevalence of repeats tends to be lower in MII oocytes and zygotes compared to late-stage embryos, indicating repeat activities alone cannot entirely account for the widespread distal H3K4me3 peaks observed in these cells.

As mentioned previously, dynamic changes in H3K4me3 in humans differ from those observed in mice. H3K4me3 levels steadily decrease from the GV stage to the MII (metaphase II) stage and increase from the PN (pronuclei)-2 zygote stage. During the human embryo pre-ZGA (4-cell stage), broader marks of H3K4me3 are already evident. Overall, 53% of these marks persist and become active in the 8-cell stage, while the remaining 47%, where H3K4me3 is lost, are in the promoters of development and differentiation genes [68]. These genes remain inactive during ZGA. Compared with promoter regions, weaker but widely distributed distal marks of H3K4me3 are evident in pre-ZGA embryos, indicating de novo deposition of H3K4me3 [17]. Many of these marks of H3K4me3 overlap with regulatory elements and exhibit high chromatin accessibility at the 4-cell stage. These distal marks decrease in the 8-cell stage and are deposited in CpG-rich and hypomethylated regions [76].

The interplay between H3K4me3 and DNA methylation is pivotal for understanding genome-wide regulation. Demonstrably, DNA methylation in the maternal genome exhibits an inverse correlation with ncH3K4me3. The evidence indicates that oocytes featuring ncH3K4me3 display approximately 18% CpG methylation, starkly contrasting to the 57% observed in oocytes lacking ncH3K4me3 [12]. A significant correlation is also observed between H3K4me3 and CpG density in four-cell embryos, particularly for promoters with moderate CpG levels, showcasing transcriptional activity in post-ZGA stages [77]. H3K4me3 is recognized for its role in counteracting DNA methylation, along with repressive histone modifications such as Histone H3 Lysine 9 trimethylation (H3K9me3) and Histone H3 Lysine 27 trimethylation (H3K27me3) [56]. This interaction highlights the intricate regulatory mechanisms involved in gene expression. These bivalent domains play a crucial role in governing gene expression by maintaining a balance in the methylation levels of two histone proteins with opposing effects. This equilibrium enables them to remain suppressed, ready for activation without differentiation signals [78].

Another intriguing aspect of H3K4me3 dynamics involves cooperation with the histone variant H3.3, a crucial player after fertilization essential for embryo development. H3.3 turnover is associated with changes in histone modifications like H3K27me3 and H3K36me3 [79]. The specific correlation between H3.3 and ncH3K4me3 remains unclear, and further investigation is required to determine if H3.3 replacement is responsible for the removal of ncH3K4me3.

While the mechanism of H3K4me3 reprogramming remains an enigma, recent studies suggest that the Histone–lysine N-methyltransferase 2 (KMT2) complex may catalyze the establishment of broad H3K4me3 domains [5,80]. Identifying the transcription factors involved in this precisely controlled process is crucial for further exploration. These major reprogramming events of both promoter and distal H3K4me3 marks offer invaluable insights into the parental-to-zygotic transition in human pre-implantation embryos, providing a deeper understanding of early embryonic development and epigenetic regulation.

## 5. Unveiling H3K9me3: Orchestrating Epigenetic Landscapes in Development

H3K9me3 emerges as a sentinel, staunchly guarding the integrity of heterochromatin—a densely compacted chromatin state refractory to transcriptional activities [81,82]. This histone modification, characterized by adding three methyl groups to lysine 9 of histone H3, plays a pivotal role in orchestrating cellular processes by imposing a formidable barrier to cell fate transitions.

The functional significance of H3K9me3 lies in its ability to occlude DNA, rendering it inaccessible for transcription factors—a phenomenon intricately linked to the hindrance of the transcriptional machinery [83]. This repressive chromatin environment, governed by H3K9me3, is a regulatory mechanism controlling gene expression patterns critical for cellular identity and function.

Recent revelations from investigations into Somatic Cell Nuclear Transfer (SCNT) embryos add a layer of complexity to our understanding of H3K9me3 dynamics [84,85]. SCNT enables animal resurrection and the treatment of human diseases by reprogramming somatic cells into pluripotent states. In this context, a method fraught with challenges in achieving efficient reprogramming, the nuanced role of H3K9me3 comes to the fore [86,87,88]. Unlike fertilized embryos, SCNT embryos manifest a distinctive profile marked by gradual and incomplete demethylation of H3K9me2 and H3K9me3 [89,90]. This aberrant pattern significantly contributes to the failure of ZGA, highlighting the indispensable role of H3K9me3 in orchestrating the intricate balance between pluripotency and cellular differentiation.

### Navigating Reprogramming Challenges and Zygotic Genome Activation

The dynamic features of H3K9me3 during early embryonic development are critical for natural reprogramming post-fertilization. It is remarkable that near-exclusive expression of the H3K9me3 demethylase named Lysine-specific demethylase 4A (KDM4D), is observed in MII oocytes and has been proven to be crucial for maintaining genomic stability and ZGA [91]. However, H3K9me3 emerges as a barrier in SCNT embryos, hindering proper ZGA and compromising development [92]. Studies indicate that H3K9me3 in donor cells obstructs somatic cell reprogramming, leading to abnormal ZGA in 2-cell SCNT embryos [93,94]. At this stage, regions resistant to reprogramming (RRRs) marked by H3K9me3 impede successful reprogramming, prompting strategies to overcome these challenges.

For this reason, overexpressing KDM4D in embryos or knocking down H3K9me3 methyltransferases (Suv39h1/h2) in donor cells proves effective in rescuing ZGA-related gene transcription and enhancing blastocyst development rates in SCNT embryos [85]. It should be noted that indiscriminate depletion of H3K9me3 can interfere with the lineage-specific deposition in SCNT blastocysts, highlighting the complexity of this process. Identifying new critical regulators involved in lineage-specific H3K9me3 deposition will add a new dimension to understanding the intricacies of epigenetic reprogramming challenges.

The regulatory influence of H3K9me3 extends to impact the expression of repeat elements and protein-coding genes in mouse pre-implantation embryos [82]. Early embryonic development triggers extensive demethylation of the mouse genome, leading to a significant fraction of long terminal repeats (LTRs) becoming hypomethylated and actively transcribed, a process crucial for normal development. Post-fertilization, H3K9me3 marks within LTR retrotransposons gradually increase during pre-implantation development (Figure 1B), with the Chromatin assembly factor 1 subunit A (CHAF1A) complex playing a pivotal role in blastocyst formation and cell fate decisions [89]. Properly regulating these LTRs is essential in later developmental stages to maintain genome stability [95]. Studies in mouse embryonic stem cells (mESCs) have suggested that multiple H3K9me3 modifiers, such as Suv39h1/h2 and ERG-associated protein with SET domain (ESET) complexes, are responsible for retrovirus silencing via the establishment of H3K9me3 modifications [96,97].

In the post-implantation stage, a dynamic interplay of H3K9me3 marks re-establishes in promoter regions, orchestrating the repression of lineage-specific genes [89]. Transcription factors such as Pou5f1, Sox12, and Zfp105 contribute to epiblast-specific H3K9me3, while Zbed6, Elf4, and Glis2 are involved in extraembryonic-specific H3K9me3 formation [68]. Mutant studies underscore the significance of precisely regulated H3K9me3 in ensuring proper embryonic development [83]. This multifaceted role of H3K9me3 emphasizes its complex involvement in reprogramming challenges and orchestrating crucial developmental events.

## 6. H3K27me3 and H3K27ac: Dual Epigenetic Players in Zygotic Genome Activation

In the intricate landscape of epigenetic regulation governing ZGA, two histone modifications stand out as key orchestrators—Histone 3 Lysine 27 trimethylation (H3K27me3) and acetylation (H3K27ac) [4,40]. These dual epigenetic players engage in a dynamic interplay, shaping the transcriptional destiny of critical genes during the pivotal events of early embryonic development.

### 6.1. Individual Roles of H3K27me3

**Figure 2:**
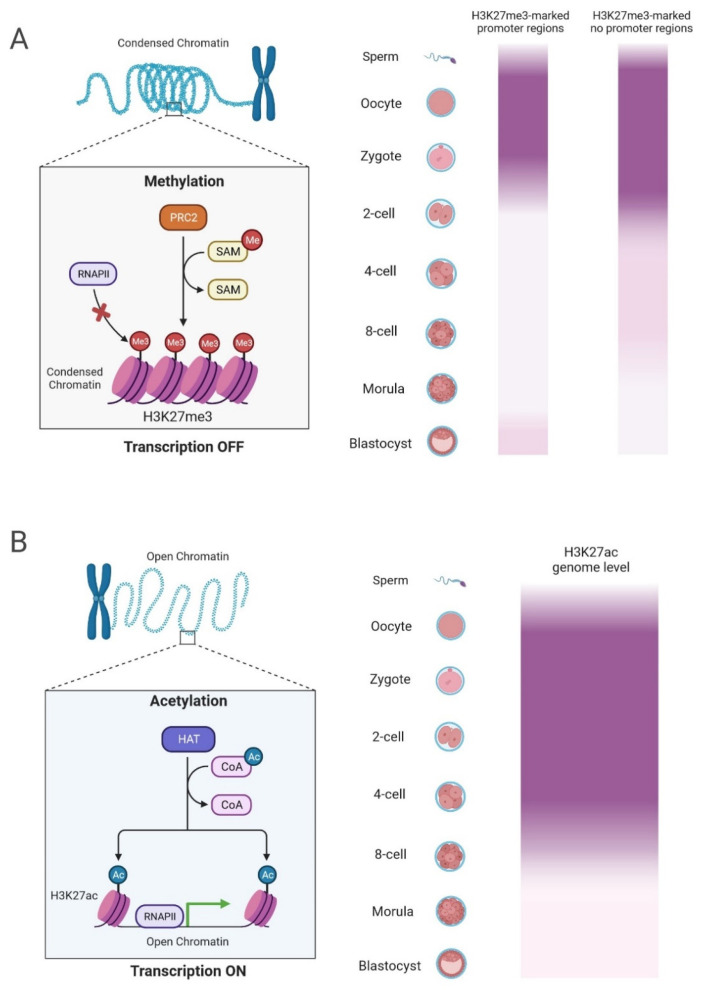
Antagonistic activity between H3K27me3 and H3K27ac in ZGA gene expression. (**A**) A polycomb repressive complex (PRC2) orchestrates the trimethylation of histone 3 on lysine 27 via histone methyltransferase activity. The SAM cofactor in Enhancer of zeste homolog 2 (EZH2), one of the PCR2 subunits, is essential to recognize the H3K27 tail selectively. The introduction of the trimethylation mark on lysine residue located at position 27 of the histone H3 tail results in the downregulation of nearby genes by forming heterochromatic regions. H3K27me3 experiences widespread loss in promoter regions at the 2-cell stage while manifesting in non-promoter regions in a highly pervasive manner. (**B**) A HAT facilitates the acetylation of histone 3 on lysine 27 by transferring an acetyl group from acetyl CoA. H3K27ac is an active enhancer mark, promoting a more accessible chromatin structure. It is present in both distal and proximal regions of genes, with enrichment observed at TSS. The H3K27ac marks exhibit discernible intensity in zygotes, 2-cell, and 4-cell embryos but gradually decline in 8-cell embryos. Abbreviations: Ac, acetylation; HAT, histone acetyltransferase; Me, methylation; PRC2, Polycomb Repressive Complex 2; RNAPII, RNA polymerase II; SAM, S-adenosylmethionine; TSS, transcriptional start sites. Created with Created with www.biorender.com (accessed on 25 December 2023).

H3K27me3 is a well-known repressive histone modification associated with gene silencing, primarily within facultative heterochromatin [98]. This modification involves adding three methyl groups to lysine 27 of histone H3 and is catalyzed by Polycomb Repressive Complex 2 (PRC2) [99,100] (Figure 2A). Playing a vital role in maintaining cellular identity by repressing genes associated with alternative cell fates, the dynamics of H3K27me3 during mammalian ZGA are crucial for unraveling the transition from a transcriptionally silent state to the activation of specific gene loci [4,101]. Recent studies have unveiled the intricate interplay between H3K27me3 and other histone modifications [78,102], shedding light on regulatory networks governing early embryonic development.

Examining H3K27 methylation dynamics during minor and major ZGA in mouse embryos reveals valuable insights [24]. The di-methylation and tri-methylation of H3K27 emerge as pivotal players in transcriptional silencing. H3K27me2, exclusively and robustly expressed in the female pronucleus during PN stages 2–3 and 4–5, significantly increases from 2- to 8-cell stage embryos, reaching its highest point in early blastocysts [103]. In contrast, H3K27me3 exhibits distinct patterns, which are prominent in the female pronucleus during PN stages 2–3 and detectable in both male and female pronuclei during PN stages 4–5 [104]. A large-scale loss of H3K27me3 in promoter regions occurs from PN-5 onward [105]. This loss involves global erasure in the paternal genome and selective depletion in the maternal genome’s promoter regions [106,107]. Weakened H3K27me3 expression is already evident in 2-cell stage embryos. It intensifies later in 4-cell stage embryos, diminishes in 8-cell stage embryos, and becomes barely detectable in early blastocysts (Figure 2A). The dynamic fluctuations in H3K27me2 and H3K27me3 offer critical insights into their roles throughout the early stages of embryonic development [108], strongly suggesting their involvement in the intricate regulation of gene expression and chromatin accessibility.

In human embryonic development, H3K27me3 dynamics differ from those in mice. Notably, in human GV oocytes, H3K27me3 is selectively deposited in promoters of developmental genes and partially methylated domains, deviating from the pattern observed in mouse oocytes [109]. This initial divergence sets the stage for further discrepancies during human pre-implantation embryo development, where the resetting of H3K27me3 follows a unique trajectory compared to its counterpart in mice. Specifically, human embryos at ZGA (8-cell stage) manifest a striking absence of H3K27me3 signals [37], indicating a comprehensive erasure of this histone modification on both parental genomes. The correlation between the lack of core components of PRC2 in human embryos and the concurrent loss of H3K27me3 adds an intriguing layer to understanding epigenetic regulation [110]. This distinct scenario raises questions about regulatory mechanisms, such as the absence of imprinting regulation like X chromosome inactivation, which plays a pivotal role during early mouse embryogenesis.

Furthermore, the divergence continues as H3K27me3-mediated imprinted genes are identified in early mouse embryos, and human orthologs seem to undergo de novo deposition of H3K27me3 in humans [98,111]. However, verifying the existence of H3K27me3-controlled imprinting in early human embryos necessitates further investigation. Further analysis reveals asymmetric H3K27me3 patterning between ICM- and TE-specific genes [112,113], hinting at potential preferential deposition between distinct cell types. These intricate dynamics of H3K27me3 provide a nuanced understanding of its roles in regulating gene expression, chromatin accessibility, and imprinting during the complex process of embryonic development.

Building upon the dynamics of H3K27me3 in early embryos, Guo H et al. have reported a strong negative correlation between H3K27me3 enrichment and DNA methylation of promoter regions of genes in MII oocytes, suggesting a complex interplay between these epigenetic marks during early development [13]. However, noncanonical H3K27me3 marks, pervasive in non-promoter regions [114], raise questions about their functional significance and the molecular mechanisms governing these patterns.

### 6.2. Distinctive Functions of H3K27ac in ZGA

In stark contrast with H3K27m3, H3K27ac is generally associated with active transcription [57]. Catalyzed by histone acetyltransferases (HATs), adding acetyl groups to lysine 27 of histone H3 induces a more open chromatin structure, facilitating the binding of transcription factors and promoting gene expression (Figure 2B). As the zygotic genome undergoes activation, the presence of H3K27ac at specific genomic loci marks regions poised for transcriptional activity, a crucial indicator for the timely and precise expression of genes directing cell fate decisions and embryonic development [115].

Recent studies in mice have explored the existence of broad H3K27ac domains in embryos before the ZGA stage [116]. These investigations revealed that H3K27ac signals cover 17.6% of the mouse genome in zygotes, but this coverage drops below 10% in post-ZGA embryos. Broad domains (>10 kb) of H3K27ac are widely detected in PN-5 zygotes, some exceeding 20 kb. In contrast, broad domains >20 kb become limited in mouse embryos at the 2-cell and 8-cell stage [40].

Comparing H3K27ac-enriched regions between mouse gametes and zygotes reveals a higher inheritance of these marks from oocytes than those transmitted by spermatozoa. The data underscore the significant resemblance in the distribution of the H3K27ac signal across the entire genome of the mouse zygote to that of the mouse MII oocyte, in contrast to sperm and early post-ZGA embryos. Moreover, a significant expansion of H3K27ac domains, exceeding a length of 20 kb, is observed in the zygote compared to oocytes and sperm, suggesting a reprogramming of the H3K27ac pattern after fertilization [40].

In the mouse zygote, 68.3% of narrow H3K27ac peaks, with lengths less than 10 kb, originate de novo in regions without H3K27ac enrichment in mouse gametes. Most broad H3K27ac domains (>10 kb) are established de novo by extending H3K27ac peaks in gametes post-fertilization. Of these domains, 8.0% are exclusively inherited from the oocyte, 4.8% are from the spermatozoa, and 0.5% are from shared broad domains between the oocyte and sperm. An additional comparison between H3K27ac patterns in spermatozoa and the human 2-cell embryo reveals that merely 0.4% of broad H3K27ac domains in the human 2-cell embryo are inherited from spermatozoa. Primarily, these broad H3K27ac domains in human 2-cell embryos do not originate from sperm [40].

Exploring the dynamics of histone H3K27ac in early human embryos unravels intriguing patterns. Utilizing an efficient ChIP-seq method, these studies demonstrate a genomic distribution of broad H3K27ac domains spanning over 10 kb in 2-cell and 4-cell embryos [57], with some domains exceeding 50 kb [40]. These broad domains markedly decrease in 8-cell embryos and subsequent stages, suggesting an association with zygotic genome activation in humans. Notably, robust H3K27ac signal intensity is observed in zygotes, 2-cell, and 4-cell embryos before decreasing in 8-cell embryos (Figure 2B), supporting the observation of broad domains in the earliest stages of development.

Furthermore, intergenic and intronic regions exhibiting a prevalence of H3K27ac enrichment have been identified in early embryos. The H3K27ac signal spreads across the promoters of protein-coding genes at the 2- and 4-cell stages while being depleted at TSSs. However, after the 8-cell stage, the H3K27ac signal concentrates around the TSS [40]. Interestingly, most promoters with H3K27ac at the zygotic genome activation stage exhibit broad H3K27ac domains before this activation [8]. The association between broad H3K27ac domains and partially methylated domains (PMDs) suggests a dynamic interplay between H3K27ac marks and DNA methylation during the early stages of human embryonic development [117]. In particular, promoters covered by broad H3K27ac domains show higher CpG densities than those without a broad H3K27ac signal [118].

The dynamics of typical H3K27ac peaks during early embryogenesis reveal significant changes at the 8-cell stage. Broad H3K27ac domains transition to typical H3K27ac peaks in human embryos during this phase. A detailed analysis unveils that approximately 80% of H3K27ac peaks are in distal regions, i.e., non-promoter regions, at the 8-cell stage. Nevertheless, over 70% of human promoters exhibit H3K27ac enrichment at this stage [40]. Studies also confirm high expression in genes whose promoters are marked with H3K27ac and/or combined with the H3K4me3 modification [57,119]. Throughout early development, from the 8-cell stage to the 6-week stage, H3K27ac peaks in promoter regions exhibit greater stability than distal H3K27ac peaks, and the number of promoters marked with H3K4me3 undergoes minimal changes. Despite this stability, many stage-specific genes are expressed during the morula, blastocyst, and 6-week embryos, and this expression is correlated with stage-specific modifications of H3K27ac in promoters [40]. These findings reinforce the notion that establishing H3K27ac modification in promoters plays a crucial role in the temporal regulation of gene expression during human embryogenesis.

## 7. Dynamics of Other Histone Modifications in Early Embryonic Development

### 7.1. H3K36me3 Dynamics Unveiled: Allelic Reprogramming in Early Mouse Embryos

The methylation of H3K36 (H3K36me), a highly conserved process from yeast to humans, is intricately associated with transcribed regions, playing pivotal roles in transcription fidelity [120,121], RNA splicing [122,123] and DNA repair [124,125]. In mammals, SET domain containing 2 (SETD2) emerges as the primary methyltransferase responsible for catalyzing H3K36 trimethylation (H3K36me3) in vivo [126,127,128]. SETD2 facilitates the interaction with RNA polymerase II, orchestrating the coupling of H3K36me3 with transcription elongation [129]. Unlike H3K4me3, H3K36me3 exhibits a positive correlation with DNA methylation, recruiting DNA methyltransferase 3A and 3B (DNMT3A/B) and maintaining this association in various mammalian cells [130,131].

Several studies underscore the critical roles of SETD2 levels and H3K36me3 in establishing and safeguarding the maternal DNA methylome during oogenesis and early embryo development. In mice, SETD2-depleted oocytes experience a significant loss of H3K36me3, leading to invasions of H3K4me3 and H3K27me3 into regions formerly marked by H3K36me3 [68]. Additionally, SETD2-deficient oocytes result in an aberrant DNA methylome characterized by the loss of maternal imprints and anomalous deposition of H3K4me3 instead of DNA methylation, particularly at imprinted control regions (ICRs) [132].

Furthermore, the scarcity of SETD2 has been demonstrated to induce defects in oocyte maturation and embryonic lethality. Mice deficient in SETD2 do not survive beyond embryonic day (E) 10.5–E11.5 [133]. Notably, the overexpression of H3.3K36M (lysine to methionine mutant) in mouse MII oocytes results in reduced H3K36me3 levels and compromised embryo viability [134].

The study of allelic reprogramming of H3K36me3 post-fertilization in early mouse embryos has provided valuable insights. Given the transient inheritance of maternal marks H3K4me3 and H3K27me3, influencing processes like ZGA [56], imprinted X chromosome inactivation [135], and gene expression [136], the inquiry arises regarding the inheritance of H3K36me3 in early embryos and its potential interaction with other epigenetic marks. Recent immunofluorescence studies have uncovered the presence of H3K36me3 at all stages except in paternal pronuclei shortly after fertilization [132]. Analyses of H3K36me3 via ChIP-seq in sperm and discrimination of parental strains via single-nucleotide polymorphisms (SNPs) revealed a significant allelic imbalance in 1-cell embryos, with a notably higher number of maternal reads than paternal reads [137,138]. Surprisingly, H3K36me3 inherited from oocytes appears present in 1-cell embryos but diminishes considerably by the late 2-cell stage and is lost by the 8-cell stage [139]. Conversely, most, if not all, H3K36me3 peaks in sperm are lost in zygotes [76]. The temporal transition of H3K36me3 from parental to zygotic patterns aligns closely with ZGA, suggesting allelic reprogramming during early embryo development [140]. Notably, maternal H3K27me3 persists beyond ZGA in the blastocyst, potentially influencing the deposition of zygotic H3K36me3. Genes with paternal-specific H3K36me3, as opposed to maternal-specific H3K36me3, exhibit a preference for reciprocal allelic H3K27me3. This group includes many H3K27me3-controlled imprinted genes, indicating the occurrence of H3K36me3 in early embryos and its role in marking allele-specific gene expression [68].

### 7.2. Histone H3R26me2: Pivotal in Cell Fate Determination in Embryos

After ZGA, embryos undergo multiple cell divisions before the first segregation into cell lineages, giving rise to TE and ICM cells. TE lineage, marked by Cdx2 and Gata3 expression, plays a role in placental development [141,142,143]. In contrast, cells in the ICM, recognized by pluripotent factors, undergo differentiation into epiblast and primitive endoderm, giving rise to all embryonic tissues and certain extraembryonic membranes [144,145,146]. During differentiation, the HIPPO pathway regulates the TE lineage [147,148], and epigenetic modifications consolidate the ICM lineage [149].

Previously explored studies have highlighted the role of Histone 3 Arginine 26 dimethylation (H3R26me2) as a recently identified epigenetic mechanism [150]. This process, predominantly governed by Coactivator-associated arginine methyltransferase 1 (CARM1), has been reported to influence pluripotency in mouse embryos and mESCs [151]. The overexpression of CARM1 in embryonic stem cells (ESC) and early mouse embryos’ blastomeres drives an increase in H3R26me2 at pluripotent gene promoters [152,153,154], such as Oct4/Pou5f1 [155], Nanog [156], and Sox2 [157,158]. This epigenetic mark linked to gene activation determines the elevated expression of these genes, closely related to cell fate determination and the pluripotent capacity of these cells.

The asymmetrical distribution of H3R26me2 in blastomeres is detected in embryos as early as the four-cell stage [153]. Blastomeres with higher levels of CARM1 and H3R26me2 contribute more significantly to the formation of the ICM [159]. Furthermore, higher levels of H3R26me2 enhance the expression of pluripotent genes and facilitate Sox2 binding to its targets, contributing to ICM specification. Additionally, CARM1 overexpression can increase the frequency of asymmetric cell divisions, leading cells to adopt a more internal position in the embryo [159]. Alongside CARM1, another chromatin regulator named PR domain-containing 14 (PRDM14) is also asymmetrically expressed in four-cell embryos, thus modulating the level of H3R26me2 to favor contribution to the ICM [160,161,162].

Early cell fate determination in mouse embryos has recently been suggested to commence at the late 2-cell stage [163]. At this point, a long non-coding RNA (lncRNA) known as LincGET is transiently and asymmetrically expressed in the nucleus, extending from the 2-cell to the 4-cell stage [155]. LincGET interacts with CARM1, accumulating it in nuclear granules that require the presence of the Nuclear Paraspeckle Assembly Transcript 1 (NEAT1) and its partner Nuclear RNA-binding protein 54 kDa (P54NRB) [163,164]. It results in a significant increase in the H3R26me2 level, activation of ICM-specific gene expression, positive regulation of transposons, and an increase in global chromatin accessibility. It is important to note that introducing LincGET into one of the blastomeres of 2-cell embryos can potentially redirect their differentiation toward the ICM [155].

The lncRNA named Neat1 is also required to mark H3R36me2, which is CARM1-dependent and crucial for ICM specification [165]. NEAT1 shows an asymmetrical expression among blastomeres in 4-cell embryos and recruits CARM1 in paraspeckle nuclear foci [140]. Disruption of NEAT1 results in a decrease in H3R26me2, an increase in Cdx2 expression, and a biased specification toward the TE lineage [153,166].

In summary, the asymmetry in the distribution of H3R26me2 emerges as one of the early signals guiding lineage specification. The variability in H3R26me2 is carefully regulated by the asymmetrical expression of CARM1, PRDM14, LincGET, and NEAT1 in the early blastomeres. Although the cause of the initial skewed expression of CARM1/PRDM14/LincGET/NEAT1 in 2-cell and 4-cell embryos is not fully understood, these findings provide a clearer understanding of the molecular events orchestrating cell fate determination in the early stages of embryonic development.

## 8. Functional Diversity of Histone Variants in the Activation of the Zygotic Genome

The histone variants exhibit distinct positioning and dynamics within cells, assembling into nucleosomes via different molecular chaperones. They interact with various chromatin remodeling complexes, replacing canonical histones or undergoing substitution with other variants during cellular development and differentiation [167,168,169]. Structural differences introduced by a central histone variant can impact histone interactions, transforming nucleosome stability and chromatin opening or compaction [170]. Among these, histone H2A variants are recognized for coordinating early embryonic genome chromatin remodeling by replacing conventional H2A in a subset of nucleosomes [171,172].

The macroH2A histone variant is a central histone related to canonical H2A, possessing a long non-histone domain (NHD) at the C-terminus [173]. Previous studies have implicated macroH2A in epigenetic gene silencing events, including X chromosome inactivation [174,175]. However, macroH2A1 is expressed at similar levels in both male and female cells [176], suggesting its function extends beyond X chromosome inactivation. Further analyses have revealed that macroH2A can inhibit transcription by negatively regulating the binding of the NF-kappaB transcription factor and preventing SWI/SNF chromatin remodeling) [177,178,179]. MacroH2A is localized in the chromatin of GV oocytes, associated with mature oocyte chromosomes, and abundant in the first polar body. After fertilization, a transient asymmetry is observed, with macroH2A preferentially associating with the female pronucleus. This maternal reserve of macroH2A is depleted by the late PN-2 stage, resulting in embryos at the 2-, 4-, and 8-cell stages lacking macroH2A, except in residual polar bodies [177,180]. This depletion supports the repressive effect of the macroH2A variant, as it must be removed before ZGA to enable the early embryonic cells to become transcriptionally active [7,181]. MacroH2A protein expression reappears in embryos after the 8-cell stage and persists in morula and blastocysts, where nuclear macroH2A is present in both trophectodermal cells and the inner cell mass [177]. This finding suggests that embryos complete their initial three- or four-cell cycles without macroH2A. These findings imply significant modifications in macroH2A variant content in the chromatin of developing embryos before implantation.

Another identified H2A variant is H2A.X, which plays a role in DNA repair [182,183]. In mammals, H2A.X shares up to 95% sequence similarities with canonical H2A and is highly conserved across species [184]. H2A.X contains a unique SQ motif at its C-terminus and is invariant in sequence and position relative to its C-terminus across species [185,186]. Recent studies have demonstrated that H2A.X regulates Cdx2 and its specific extraembryonic genes, determining the developmental potential of stem cells [187,188] and indicating regulatory functions of H2A.X in the transcriptional network related to cellular fate control.

H2A.X is the main H2A variant deposited on chromatin in cleavage-stage embryos in mice and humans with ZGA activity. This histone variant is specifically expressed in 1–2 cell stage mouse embryos [189] and shows an enrichment trend in human embryos at the 4–8 cell cleavage stage [170]. The proper amount of H2A.X ensures that genes involved in ZGA are at relatively normal expression levels. A recent study in embryonic stem cells (ESCs) identified that H2A.X inhibits the expression of genes mediated by Dux [190,191], a factor directly involved in ZGA stimulation, by binding to its locus, confirming that the dynamic incorporation of this histone variant finely modulates developmental progression.

The H2A.Z histone variant from yeast to mammals constitutes approximately 4 to 10% of total H2A histones [192]. Its multifaceted role includes crucial functions in transcriptional control [193], DNA repair [194], heterochromatin formation [195,196], and genetic stability [197,198]. Genomically, H2A.Z integrates into chromatin, playing an essential regulatory role in transcription. Despite its functional relevance, studies on H2A.Z in early developmental stages have been constrained by the lethality accompanying its mutation in various organisms [199,200,201]. However, multiple studies concur that it plays a crucial role as a regulator in the activation and transcription of genes during ZGA [192,202].

Two isoforms of H2A.Z have been identified, differing in only three amino acids. These variants, H2A.Z.1 and H2A.Z.2, are encoded by separate genes, H2AFZ and H2AFV, respectively [198]. Despite the subtle difference in three amino acids between these isoforms, they perform specialized functions related to their interactions. While H2A.Z.2 preferentially associates with H3K4me3, it has been confirmed that H2A.Z.1 interacts more efficiently with Bromodomain-containing protein 2 (BRD2) [193,203,204].

The deposition of H2A.Z on the TSS of the zygotic genome is facilitated by an ATPase chaperone known as Domino in *Drosophila* [205]. This deposition precedes ZGA and RNA polymerase II (Pol II) binding to chromatin, indicating its contribution to preparing genes for transcriptional activation [204]. Although the mammalian orthologs of Domino, Snf2 Related CREBBP Activator Protein (SRCAP), and E1A Binding Protein P400 (EP400) [206] have not been fully explored during early embryogenesis, previous studies have shown that EP400 is essential for the identity of ESCs [207], and EP400 mutant mice are lethal when homozygous [208]. This observation underscores the need for further research to better understand the dynamics of SRCAP and EP400 in the context of early developmental regulation.

During minor ZGA, H2A.Z is symmetrically expressed in male and female pronuclei in embryos at PN 2–3 and PN 4–5. However, variant expression slightly decreases in embryos at the 2-cell stage, reaching a higher level in embryos at the 4-cell stage. In embryos at the 8-cell stage and early blastocyst, the expression level of H2A.Z decreases, suggesting a temporal regulation of its function during later stages of embryonic development [209]. It has been verified that H2A.Z deposited by Domino in *Drosophila* and its mammalian orthologs, provided by the mother, are necessary for the transcriptional activation of thousands of genes at the onset of ZGA; the lack of expression of these, including regulators of this process, leads to embryonic death [192]. In mESCs, H2A.Z in chromatin is linked to H3K4me3, present in both active and bivalent promoters but not in repressed genes [202]. This correlation pattern is maintained in human embryonic stem cells (hESCs) [210]. These findings expand our understanding of ZGA regulation, emphasizing the importance of chromatin in this process. Given the evolutionary conservation of H2A.Z and the fundamental principles of ZGA, it is speculated that histone variants could play similar roles during mammalian embryogenesis. Future research in this direction will illuminate the complex process by which chromatin states and transcription factors jointly orchestrate zygotic genome activation.

Finally, we encounter the H3.3 variant, which has sparked considerable interest due to its distinctive role in remodeling the male and female genomes during fertilization and the early stages of embryonic development. This histone plays a vital function in maintaining genomic integrity in mammals [211]. Encoded by two different genes, *H3F3A* and *H3F3B*, H3.3 generates an identical protein product [212]. Its constitutive expression in cells and its incorporation into chromatin independently of DNA synthesis underscore its relevance in the biological context. In the case of mice, ZGA is associated with the extensive incorporation of the H3.3 variant into parental genomes [213]. Both in sperm and oocytes, H3.3 is enriched, with mature oocytes being particularly rich in H3 mRNA, leading to the formation of maternal H3.3 after activation. This histone also plays a crucial role in forming the male pronucleus during fertilization [214].

A detailed study using a mouse model marked with H3.3B-HA reveals the asynchronous activation of paternal and maternal genomes [79]. The early deposition of paternal H3.3 in the zygotic genome contrasts with the delay in maternal deposition until the four-cell stage. Maternally stored H3.3 in oocytes is essential for cleavage and the minor wave of ZGA [215]. Its global deposition in the paternal genome during the transition from protamine to histone [216], with a preferential enrichment in CpG-rich TSS, highlights its critical role. Depletion of maternal H3.3 can result in the loss of H3K27ac, leading to failure in the minor ZGA and early embryo arrest [217]. Mechanically, the deposition of maternal H3.3 in the sperm genome removes repressive histone modifications, promotes the establishment of active modifications, and, in turn, enables the initiation of the minor ZGA of the paternal genome. In summary, current findings emphasize that paternal chromatin remodeling mediated by H3.3 is essential for developing pre-implantation embryos and activating the paternal genome during embryogenesis, providing valuable insights into fundamental biological processes.

## 9. Future Perspectives and Applications of ZGA Histone Modifications

Our review provides a detailed insight into the dynamics of histone modifications during ZGA in mammals, highlighting various promising avenues as targets for application in the biological context, particularly in regenerative medicine. Human pluripotent cells, including hESCs and induced pluripotent stem cells (iPSCs), offer significant potential for advancements in this field. They serve as valuable models for exploring the mechanisms underlying human development and disease while presenting an opportunity as a source of replacement cells in cellular transplantation approaches. The unique chromatin marking and epigenomic patterns indicate the remarkable developmental plasticity of pluripotent cells. All major features of the hESC epigenome are re-established in iPSCs [218]. These epigenetic features are crucial for organizing genetic information to support both the maintenance of self-renewal programs and the preservation of multilineage differentiation potential [219]. Ultimately, understanding histone dynamics during ZGA could be crucial for refining the current differentiation protocols of hESCs and iPSCs, deriving well-defined, safer, and more suitable cells for replacement therapies.

Histone modifications also provide an opportunity to address potential therapeutic approaches for epigenetic regulation in reproductive medicine. Understanding how histones influence gene expression during the early stages of embryonic development not only provides valuable insights into infertility but also opens the door to optimizing assisted reproduction techniques. Precise manipulation of histones could be key to improving the quality of embryos cultured in vitro, potentially resulting in higher success rates in IVF/ICSI procedures [220]. Furthermore, this knowledge could have applications in selecting embryos for implantation, using specific histone marks as biomarkers to assess the quality and prognosis of implantation for the selected embryos. These promising applications point toward a future where a detailed understanding of epigenetic regulation could significantly transform the practice of reproductive medicine.

Another promising research direction is exploring specific transcription factors and cofactors orchestrating the reprogramming of other histones during ZGA. This approach would shed light on the molecular actors driving this critical process and could have applications in both fundamental understanding of biology and future therapeutic developments. Furthermore, conducting a comparative analysis of the dynamics of studied histones across different species could provide valuable evolutionary insights into the role of these modifications in early embryonic development, offering a broader understanding of comparative biology. Finally, understanding the specific mechanisms of histone reprogramming during early embryonic development in embryos produced by SCNT not only presents itself as a fundamental model for basic research but also paves the way for innovative approaches in animal cloning, regenerative medicine, and treating human diseases.

## 10. Conclusions

The in-depth analysis of ZGA accentuates the intricacies of this essential process in embryonic development. This review emphasizes that, regardless of variations in embryonic development, ZGA shares highly conserved control mechanisms in mammals. These finely orchestrated mechanisms establish gene expression, shaping cellular identity and fate. The pivotal role of histone modifications, including the methylation and acetylation of specific lysine and arginine residues on H3 and their variants, significantly impacts the activation or repression of gene transcription during ZGA. A comprehensive understanding of the dynamics of histone modifications during this period unveils novel perspectives for therapeutic applications. The potential to manipulate hESC and iPSC with specific histone modifications emerges as a promising approach for developing targeted cellular therapies.

## Data Availability

Not applicable.

## Abbreviations

AML	Acute myeloid leukemia
BRD2	Bromodomain-containing protein 2
CARM1	Coactivator-associated arginine methyltransferase 1
CHAF1A	Chromatin assembly factor 1 subunit A
ESCs	Embryonic stem cells
ESET	ERG-associated protein with SET domain
EZH2	Enhancer of zeste homolog 2
GV	Germinal vesicle
HATs	Histone acetyltransferases
hESCs	Human embryonic stem cells
ICM	Inner cell mass
ICRs	Imprinted control regions
iPSCs	Induced pluripotent stem cells
KDM4D	Lysine-specific demethylase 4A
KDM6A	Lysine-specific demethylase 6A
KDM6B	Lysine-specific demethylase 6B
KMT2	Histone–lysine N-methyltransferase 2
lncRNA	Non-coding RNA
LTRs	Long terminal repeats
mESCs	Mouse embryonic stem cells
MII	Metaphase II
MSCs	Mesenchymal stem cells
MZT	Maternal-to-zygotic transition
ncRNAs	Non-coding RNAs
NEAT1	Nuclear Paraspeckle Assembly Transcript 1
NHD	Non-histone domain
ntESC	Nuclear-transfer embryonic stem cells
NURF	Nucleosome remodeling factor
P54NRB	Nuclear RNA-binding protein 54 kDa
PMDs	Partially methylated domains
PN	Pronuclei
PRC2	Polycomb Repressive Complex 2
PRDM14	PR domain-containing 14
RRRs	Regions resistant to reprogramming
SCNT	Somatic Cell Nuclear Transfer
SETD2	SET domain containing 2
SNPs	Single-nucleotide polymorphisms
TE	Trophectoderm
TSS	Transcription start site
ZGA	Zygotic genome activation
